# CAGs-Net: A Novel Adjacent-Context Network With Channel Attention Gate for 3D Brain Tumor Image Segmentation

**DOI:** 10.1155/ijbi/6656059

**Published:** 2025-08-22

**Authors:** Qianqian Ye, Yuhu Shi, Shunjie Guo

**Affiliations:** College of Information Engineering, Shanghai Maritime University, Shanghai, China

**Keywords:** adjacent-context network, brain tumor segmentation, channel attention gate, target imbalance, UNet

## Abstract

Accurate brain tumor segmentation is essential for clinical decision-making, yet remains difficult to automate. Key obstacles include the small volume of lesions, their morphological diversity, poorly defined MRI boundaries, and nonuniform intensity profiles. Furthermore, while traditional segmentation approaches often focus on intralayer relevance, they frequently underutilize the rich semantic correlations between features extracted from adjacent network layers. Concurrently, classical attention mechanisms, while effective for highlighting salient regions, often lack explicit mechanisms for directing feature refinement along specific dimensions. To solve these problems, this paper presents CAGs-Net, a novel network that progressively constructs semantic dependencies between neighboring layers in the UNet hierarchy, enabling effective integration of local and global contextual information. Meanwhile, the channel attention gate was embedded within this adjacent-context network. These gates strategically fuse shallow appearance features and deep semantic information, leveraging channel-wise relationships to refine features by recalibrating voxel spatial responses. In addition, the hybrid loss combining generalized dice loss and binary cross-entropy loss was employed to avoid severe class imbalance inherent in lesion segmentation. Therefore, CAGs-Net uniquely combines adjacent-context modeling with channel attention gates to enhance feature refinement, outperforming traditional UNet-based methods, and the experimental results demonstrated that CAGs-Net shows better segmentation performance in comparison with some state-of-the-art methods for brain tumor image segmentation.

## 1. Introduction

Gliomas represent the most prevalent primary malignant brain tumors, characterized by varying degrees of aggressiveness, heterogeneous histological subregions (including peritumoral edema, invasive tissue, necrotic core, and enhancing and nonenhancing tumor core (TC) [1]), and consequently, highly variable prognoses. Clinical MRI protocols for glioma assessment routinely incorporate multiparametric sequences—including native T1, contrast-enhanced T1 (T1ce), T2, and FLAIR—to characterize the tumor's heterogeneous substructure [2, 3]. Accurate segmentation of these tumor subregions from MRI is therefore paramount for diagnosis, surgical planning, treatment monitoring, and survival prediction [2]. Manual segmentation by radiologists, however, is labor-intensive, time-consuming, and subject to interobserver variability, driving the need for robust semiautomatic or fully automatic segmentation methods to enhance efficiency and objectivity. Despite significant advances, automatic brain tumor segmentation remains a formidable challenge. Key difficulties include the often small size and highly irregular shape of certain subregions (e.g., enhancing tumor and necrotic core), the inhomogeneous intensity distribution within tumor tissues across different MRI sequences, and the frequently poor contrast and blurred boundaries between tumor subregions and adjacent healthy brain tissue or between different subregions themselves (e.g., nonenhancing tumor vs. edema) [4].

Recently, deep learning has become a powerful alternative to image segmentation, with the ability to automatically extract and combine high distinguishable features. The features automatically extracted by deep learning are generally superior to manual feature sets and predefined feature sets [5]. As the most mainstream method of deep learning, convolutional neural network (CNN) has been widely used in brain tumor segmentation based on MRI images [6]. The CNN architecture has rapidly evolved from single-label segmentation to intensive semantic segmentation [7]. Because CNN runs on a patch with the little kernel, it has the superiority of considering context and working with raw data. For example, Menze et al. proposed an automatic segmentation method based on 2D CNN, which has been widely used in the computer vision application of natural images. This method was implemented by processing 3D medical images into 2D slices independently, such that the spatial structure of medical image data was not fully utilized [8].

Although full 3D CNN increases the number of parameters and computing requirements, 3D CNN can integrate the information of multidimensional structure and provide more information for the segmentation of lesions [9]. Meanwhile, the use of small patch size in training can reduce the memory requirement. Therefore, Kamnitsas et al. proposed the method of a two-pathway brain tumor segmentation network based on 3D CNN (named as DeepMedic) [10]. This method considers the local and global information of multimodal tumor images, but the use of multibranch full connect integration will increase the model reasoning time. Furthermore, the data flow of each branch is separated, which ignores the correlation between every layer. In order to improve efficiency, encoder and decoder structures such as full convolution network (FCN) and UNet are widely used to achieve dense prediction [11–21]. For example, Zhao et al. proposed a model by integrating FCN and conditional random fields (CRFs) to obtain the brain tumor segmentation results with appearance and spatial consistency, where multimodal brain tumor images were segmented by FCN and then smoothed by CRF [12]. Isensee et al. [13] embedded a local context module into a residual module and merged multilevel feature maps of the same channel in the decoding layer based on 3D UNet [14]. However, the correlation between adjacent layers still weakens although UNet and FCN consider the correlation between layers. Therefore, the above methods are still unable to distinguish small lesion areas, especially in the case of multiple lesion areas interweaving; the accuracy of segmentation will be greatly limited when multiple lesions interfere with each other.

In deep learning, the traditional attention mechanism highlights useful information in a feature map while suppressing irrelevant information, and it is also a tool for combining local and global responses simultaneously [22–24]. Pian et al. proposed a target classification model for visualization of the optic nerve information pathway, which confirmed the importance of the attention mechanism in CNN [15]. Schlemper et al. used the self-gate soft attention mechanism to generate end-to-end trainable gating signals, thus allowing the network to capture local information useful for prediction [16]. Oktay et al. used the attention gate to train implicitly to learn to suppress irrelevant areas in the input image while highlighting salient features useful for pancreas segmentation [17]. Sinha et al. used dual self-attention [18] to combine global dependencies to improve the generalization ability of organ segmentation [19]. However, dual self-attention is inferred only based on the self-feature map. The ability of feature representation is limited. The above traditional attention mechanism can quickly calibrate the target area, but it does not consider the direction of feature refinement.

To solve the above problems, a novel segmentation method for glioma is introduced to highlight the correlation between adjacent layers. In particular, an adjacent-context module (AdjContext-module) based on a residual module is proposed to highlight the correlation of feature maps in each hierarchy, which can semantically correlate local feature maps between adjacent layers to form global correlation information layer by layer while extracting the context feature map based on the UNet. Meanwhile, a channel attention gate (CAG) based on an attention gate is proposed to fuse rich global correlation information by making full use of the spatial information of the deep and shallow layers in the encoding layer. The attention gate as voxel attention can adaptively suppress nonlesion noise from shallow features and append lesion details to deep features. To supplement voxel space, channels are aimed at recalibrating the characteristic response of the voxel space direction. It can automatically learn a set of weights to complement and refine the features of each layer for brain tumor image segmentation networks through exploiting deep semantic features and shallow appearance features to locate each lesion subregion. Furthermore, the hybrid loss between generalized dice loss (GDL) and binary cross-entropy loss function is employed to solve the target imbalance of lesions. From the experimental results, the adjacent-context network with a CAG for brain tumor image segmentation shows well segmentation performance.

The remainder of this paper is organized as follows.  Section 2 introduces the details of the CAG network, which forms attention features by effectively utilizing the semantic features and appearance features of multilayer coding, and effectively connects the coding layer and decoding layer at the same time. In Section 3, the experimental results of three-dimensional brain glioma segmentation are presented.  Section 4 gives the conclusion of this research and discusses the proposed CAG network in detail.

## 2. Methods

It is a challenging task to segment tumor regions from multimode MRI images. In particular, the uneven intensity distribution of tumor lesion tissues in images and the poor contrast of lesion tissues are caused by the large morphological variation of glioma in MRI images. Autonomous learning of multiscale context information, regional semantics, boundary details, and other factors to obtain more discriminative characteristics of glioma is crucial for accurate and robust tumor lesion segmentation. In order to solve the above problems, we propose an AdjContext-module based on a residual model to highlight the correlation of feature maps in adjacent layers. Meanwhile, we propose a CAG with autonomous localization learning to better identify the lesion area of glioma and update the original network structure. The following sections describe the proposed approach in detail and explain the CAG module.

### 2.1. The Deep Segmentation Network With Adjacent Context

**Figure 1:**
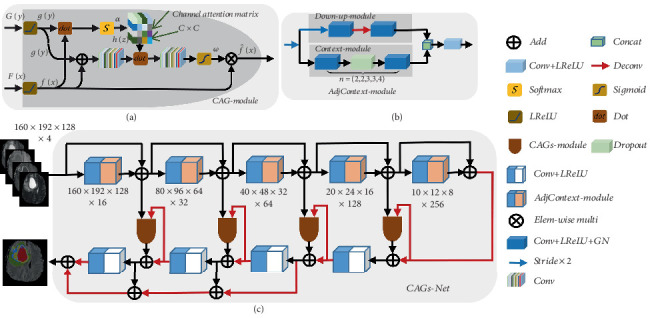
Model architecture diagram. (a) The channel attention gate module; (b) AdjContext-module; (c) CAGs-Net. The upper part of the CAGs-Net structure is used for feature extraction (which can be called encoder), and the lower part is upsampling (which can be called decoder). Firstly, the images are encoded to obtain the feature maps of each layer after the input of preprocessed images. Secondly, the feature maps of each layer are weighted by CAGs and filtered to obtain the required components for segmentation. Besides, each focused feature maps are fused with each feature map of decoding layers to obtain the feature maps that are conducive to segmentation layer by layer. Finally, results of each subregion are obtained by fusing the feature maps of the top three layers of the decoding layer.

Built on an encoder–decoder architecture, UNet produces multiresolution feature maps. The shallow feature map has high resolution and rich detail information, such as texture information. A deep feature map has low resolution and rich semantic information. Meanwhile, the residual module is used in the process of downsampling on UNet. To fuse information and global context information, we integrate the path of AdjContext-module into the residual module. AdjContext-module contains the down–up module and context module, in which the down–up module establishes the relationship between adjacent layers, and the context module extracts the context information. The convolution of numbers of Convolution + LeakyReLU + GroupNormalization (Conv + LReLU + GN) in the context module is set as *n* = (2, 2, 3, 3, 4) in each layer, respectively. The structure is shown in Figure 1b.

AdjContext-module resamples multiscale feature maps to obtain more accurate tumor expression, rather than directly convolving feature maps to extract tumor regions. As shown in Figure 1b, the feature maps of the context acquisition module and the downsampling module are combined for convolution operation to form a feature graph of context information. The AdjContext-module is composed of two convolution cores of 3 × 3 × 3 in serial convolution. In the decoding process, the last three layers are taken full advantage of as the source of generating the final feature map. The pattern of fusion features is summation to make the fusion features remarkably distinguishable.

The shallow spatial features of the network are too accurate and lack semantic information (such as color difference and contour). Although UNet integrates features of two levels through concatenation to obtain feature maps, this fusion mode may be too rough to accurately describe features of local space. Meanwhile, computational complexity is also increased in the whole reasoning process. To alleviate the large-scale changes of brain tumors and refine the characteristics of glioma images, we proposed a CAG module according to the principle of the attention mechanism. The proposed attention module combines appearance features of the current layer and semantic features of the next layer to serve as input and will generate an output attention feature map containing sparse and typical local information. The specific process is shown in Section B. The segmentation network of adjacent-context networks combined with many CAG modules is called CAGs-Net, which is shown in Figure 1c.

In our network, dropout [25] and group normalization (GN) [26] are used to prevent the training from becoming extremely overfitting, due to the constant adaptation of the network to new data distribution. GN is a standardized function, and it can increase the generalization ability of the model by calculating the mean and standard deviation of all voxels of a single image. The reason for not using batch normalization [27] is that batch normalization on a small patch will lead to significant error. Therefore, our proposed model may be trained using small batches of dataset. Furthermore, LeakyReLU (LReLU) is used as the activation function to increase the nonlinearity of the network and improve the generalization ability of the model, which can also reduce the interdependence of parameters and alleviate overfitting.

### 2.2. Adaptive CAG

Through observing the network structure of the adjacent-context network, we have found that appearance features are gradually guided to suppress details that were not in the semantic significance area and to capture more details in the semantic significance area. At the same time, appearance features can enhance their detailed features while semantic features are used to obtain the vague area of glial tumor abstraction in the deep layer so that it may lack detailed features. However, the fusion method in the adjacent-context network may be too rough to accurately describe local spatial features and global feature distribution, and the traditional attention does not consider the direction of feature refinement although it can quickly calibrate the target area. Therefore, CAG is proposed to reduce false-positive prediction of small subregions with large shape variabilities (such as enhanced tumor (ET) and TC) and to make appearance features and semantic features fully complement and refine each other, which are shown in Figure 1a. By using the proposed CAG to adaptively weight the features of each layer, our network can learn to select more discriminating features for accurate and robust glioma segmentation. This attention method can refine and merge multilevel features through the dual attention of channels and 3D space voxel information.

The attention refining features and self-filtering are generated by the proposed CAG, and the multilevel CAG and the selectively utilization of multilevel features are studied. This module can automatically learn a set of weights to represent the importance of the feature maps. The correlation operation on the input feature maps and weights itself through channel which can find direction of feature refinement. The channel attention correlation coefficients are calculated through fusing shallow appearance information and deepen global correlation semantic information. Firstly, assume that the image features of a hidden layers *x* ∈ *R*^*C*×*N*^ denotes appearance feature maps and *y* ∈ *R*^*C*×*N*^ denotes semantic feature maps, where *C* is the channel and *N* is the product of three-dimensional voxel values, and then form two feature spaces *F*, *G* by convolution, in which *N* = *w* × *h* × *l*. Next, we can obtain *F*(*x*) = *W*_*F*_∙*x*, *G*(*y*) = *W*_*G*_∙*y*, *f*(*x*) = *σ*(*F*(*x*)) ∈ *R*^*C*×*N*^, and *g*(*y*) = *σ*(*G*(*y*)) ∈ *R*^*C*×*N*^ where *W*_*F*_ ∈ *R*^*C*×*N*^ and *W*_*G*_ ∈ *R*^*C*×*N*^ are learning parameter matrix. *σ*(∙) = max(0, ∙) + *μ*∙min(0, ∙) is the LReLU function, and *μ* denotes the leakage parameter. The LReLU called unsaturated activation function is the solution of gradient disappearance.

Secondly, the correlation coefficients matrix is calculated as follows:

(1) $$\begin{array}{c} \delta =f\left(x\right)⊙g{\left(y\right)}^{T}\in R^{C\times N} \end{array}$$

where ⊙ denotes point product and then *δ* is activated through using SoftMax. Moreover, SoftMax will yield sparser activations at the output to reduce the imbalance of attention correlation coefficients matrix. Meanwhile, other attention matrix will be classified by SoftMax response computing. Thus, the direction of feature refinement becomes clearer. Then, channel focus area coefficient can be calculated as follows:

(2) $$\begin{array}{c} \alpha_{i,j}=\frac{\exp\left(\delta_{i,j}\right)}{\sum_{j}^{N}\exp\left(\delta_{i,j}\right)}\in \left[0,1\right] \end{array}$$

where *j* ∈ *C* and *C* represents the matrix of the attention which is computed by feature map between appearance information and global correlation semantic information in the *j*^th^ channel domain to the *i*^th^ channel domain. The appearance information layer filtered by attention is *r* = (*r*_1_, *r*_2_, ⋯, *r*_*i*_, ⋯, *r*_*C*_) ∈ *R*^*C*×*N*^ and *r*_*i*_ is as follows:

(3) $$\begin{array}{c} r_{i}=\overset{C}{\underset{j=1}{\sum }}\alpha_{i,j}h\left(z_{j}\right) \end{array}$$

where *h*(*z*_*j*_) ∈ *h*(*z*) and *z* = *f*(*x*) + *g*(*y*) denotes feature map after spatial information fusion. *h*(*z*) ∈ *R*^*C*×*N*^ is fusion 3D space voxel information feature maps calculated by summation, which is the source of the gate signal and can be expressed as follows:

(4) $$\begin{array}{c} h\left(z\right)=W_{h}\left[f\left(x\right)+g\left(x\right)\right] \end{array}$$

where *W*_*h*_ ∈ *R*^*C*×*N*^ are learning parameter matrix, and they are all implemented by convolution. Then, the output gate weight coefficients *ω* = (*ω*_1_, *ω*_2_, ⋯, *ω*_*i*_, ⋯, *ω*_*C*_) ∈ *R*^*C*×*N*^ can be calculated as follows:

(5) $$\begin{array}{c} \omega_{i}=\frac{1}{1+\exp\left(-r_{i}\right)}\in \left[0,1\right] \end{array}$$

where *ω*_*i*_ is the gate weights of *i*^th^ spatial voxel values transformed by a sigmoid function. The gate weight coefficients are sigmoid function response value representing the confidence coefficient that appearance feature map belongs to useful information. The larger the response value, the more likely appearance information is to be useful. It will make the useless feature response signals suppressed. The feature map *r* is the weight score calculated by a sigmoid function.

Finally, the final output layer is $\tilde{f}=\left(f_{1},f_{2},\cdots ,f_{i},\cdots ,f_{N}\right)$ calculated as follows:

(6) $$\begin{array}{c} \tilde{f}\left(x\right)=f\left(x\right)⊗\omega \end{array}$$

where ⊗ denotes an element-wise product. After using the attention gates matrix *ω* to crop useless information and noise as well as suppress feature response signals, the semantic feature maps *y* will be copied and merged to appearance feature maps *x*.

## 3. Experiment and Analysis

### 3.1. Dataset and Preprocessing

Model training and evaluation utilized the publicly available dataset from the Multimodal Brain Tumor Segmentation Challenge 2021 (BraTS 2021) [28]. This dataset comprises multi-institutional preoperative multimodal MRI scans of brain glioma patients. Each patient's data includes native T1-weighted (T1), postcontrast T1-weighted (T1ce), native T2-weighted (T2), and T2 Fluid Attenuated Inversion Recovery (T2-FLAIR) volumes, facilitating robust tumor characterization. All scans were coregistered to the same anatomical template (SRI24) [29] and resampled to 1 mm^3^. All the imaging datasets were manually segmented by one or four raters according to the same labeling protocol, and the ground truth labeling was approved by experienced neuroradiologists [3].

**Figure 2:**
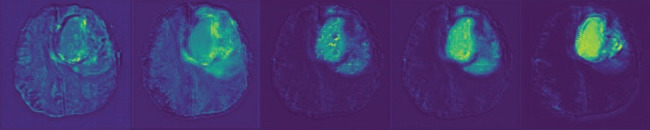
The region of attention of CAGs. The highlighted lesions are the region of the tumor subregion in which channel attention is focused.

As shown in Figure 2, subregions considered for evaluation are an ET, TC, and whole tumor (WT). ET refers to the area showing high intensity in T1CE compared with T1, and it refers to the area showing high intensity compared with healthy white matter in T1CE. TC describes the bulk of the tumor; in other words, TC includes ET, necrosis (fluid filled), and nonenhanced (solid) portions of the tumor. In T1CE, necrotic (NCR) and nonenhanced tumor (NET) cores are usually of lower intensity than in T1. WT describes the integrity of the disease because it requires TC and edema/inversion tissue (ED), which is typically a high-intensity signal in T2-FLAIR [30]. Because of these characteristics, we can segment the required lesion areas from the MRI data of the four modes of each case sample.

The dataset contains both high-grade glioma (HGG) and low-grade glioma (LGG) pathology. Moreover, MRI images may present some problems that the intensity range of the scanner is different in the same sequence. Therefore, the image data of the tumor need to be corrected for the bias field. The algorithm of N4ITK [31] is applied to bias field correction. Furthermore, due to original image data depending on the standards of instruments, normalization of the image is of critical importance. The new sample standard values *x*_*i*_ could be obtained through $x_{i}=X_{i}-\bar{X}\sqrt{DX}$, and then, labels are encoded as binary according to label classification using the one-hot method. Meanwhile, because MRI images have many background regions that do not contribute to the segmentation of lesions, the image size of 240 × 240 × 155 is cropped to 160 × 192 × 128 according to the brain distribution of MRI images, which can reduce the computational cost of the model at a certain level. Therefore, we only need to focus on the parts that are useful for segmentation in the model training, which significantly reduces the unnecessary calculation cost. Methods such as rotation, shear transformation, and random transverse shifts are used to increase the sample size in order to improve the generalization performance of the model. In our experiment, 400 images from various institutions are randomly selected as the training set and testing set, which are 320 and 80 images, respectively.

### 3.2. Implementation and Evaluation

#### 3.2.1. Loss Function

The sigmoid function is used for the output layers. The domain of the sigmoid activation function can take any range of real numbers, and the output value returned is within the range from 0 to 1, which is suitable for the segmentation of multiple subregions of brain tumors. For each of the four tumor regions (including NCR, ED, NET, and ET), we obtained a binary map with our method predictions *P* ∈ {0, 1} and experts' consensus truth *T* ∈ {0, 1}. Binary cross-entropy loss *L*_*bce*_ is a common loss function in segmentation, which is calculated as follows:

(7) $$\begin{array}{c} L_{bce}=-\frac{1}{\left|N\right|}\left[\overset{N}{\underset{i=1}{\sum }}T_{i}\log\left(P_{i}\right)+\overset{N}{\underset{i=1}{\sum }}\left(1-T_{i}\right)\log\left(1-P_{i}\right)\right] \end{array}$$

where *P*_*i*_ denotes the prediction and training output of the network and *T*_*i*_ denotes the one-hot coding of ground truth segmentation image, which has the same dimension with *P*_*i*_. A difficult point in medical image segmentation is the class imbalance of images. Using the traditional classification cross-entropy will lead to that the loss of training is impeded. Therefore, *L*_*bce*_ may lead to oversegmentation due to the class imbalance problem. The image segmentation of brain tumors, especially subregion segmentation of tumors, is a challenging multilabel segmentation task due to its shape variability and poor lesion continuity. So, the GDL [32] is also used as a loss function in medical image segmentation. The loss function *L*_*gdl*_ is defined as follows:

(8) $$\begin{array}{c} L_{gdl}=1-\frac{1}{\left|N\right|}\frac{2∙\sum_{j=1}^{M}\omega_{j}∙\sum_{i=1}^{N}P_{i,j}T_{i,j}}{\sum_{j=1}^{M}\omega_{j}∙\sum_{i=1}^{N}T_{i,j}+P_{i,j}} \end{array}$$

(9) $$\begin{array}{c} \omega_{j}=\frac{1}{\sum_{i=1}^{N}{T_{i,j}}^{2}} \end{array}$$

where the number of channels in each patch is |*N*| and *P*_*i*,*j*_ and *T*_*i*,*j*_ represent the sigmoid function output and ground truth of the *i*^th^ voxel in the *j*^th^ class, respectively. *ω*_*j*_ is used to provide the invariance of properties set of different labels, and the differentiability of *L*_*gdl*_ has been demonstrated in [32]. To solve the class imbalance problem and preserve the details on the boundary between tumor lesions, we combined *L*_*gdl*_ with *L*_*bce*_ as follows:

(10) $$\begin{array}{c} L=L_{gdl}+L_{bce} \end{array}$$

where *L* represent the loss function for model training. Binary crossover can well hinder the weakness of that GDL is biased toward large regions, and GDL can avoid binary imbalance.

#### 3.2.2. Training Process

According to the characteristics and principles of Adam [33], a high learning rate is set at the beginning, and then, the learning rate is attenuated externally with the progress of network training. This can avoid the bottleneck of model training at a certain level. The attenuation equation is as follows:

(11) $$\begin{array}{c} {lr}_{t+1}={lr}_{t}∙R^{\left(1+\tau \right)/\tau } \end{array}$$

where *lr*_*t*_ represent the *t*^th^ learning rate and *t* is the number of iterations. *R* is the learning decreasing rate, and *τ* is a threshold to trigger attenuation. The initial learning rate is *lr* = 4*e* − 5 and *R* = 0.5. The patience of learning rate is set to 100. The model is implemented with the help of GPU which uses NVIDIA Tesla V100 and Deep Neural Network library cuDNN to speed up computing.

#### 3.2.3. Evaluation Metric

In order to better measure the accuracy, robustness, and reliability of the model, the Dice similarity coefficient (DSC) [34], positive predictive value (PPV), sensitivity, and Hausdorff distance (95%) (95HD) [35] are used as the measurement indexes. DSC is adopted to evaluate the precision of tumor segmentation and measure the overlap between the manual and the automatic segmentation, which is given as follows:

(12) $$\begin{array}{c} \text{DSC}=\frac{2∙\text{TP}}{2∙\text{TP}+\text{FN}+\text{FP}} \end{array}$$

where TP denotes true positive and indicates that the original tumor region is correctly divided into tumor regions. FN denotes false negative and indicates that the originally tumor area is incorrectly classified as nontumor regions. FP denotes false positive and indicates that the original nontumor region is incorrectly segmented as a tumor region.

Furthermore, in order to measure the performance of segmentation accuracy, sensitivity is used to measure the accuracy of tumor segmentation in the misdiagnosis rate of the tumor, which can be calculated as follows:

(13) $$\begin{array}{c} \text{Sensitivity}=\frac{\text{TP}}{\text{TP}+\text{FN}} \end{array}$$

Moreover, PPV is a measure of the amount of FP and TP, and it reflects the misdiagnosis of tumor in the segmentation of nontumor regions, which is given as follows:

(14) $$\begin{array}{c} \text{PPV}=\frac{\text{TP}}{\text{TP}+\text{FP}} \end{array}$$

Finally, 95HD evaluates the distance of the edge of the tumor to the performance of the segmentation, which is computed as follows:

(15) $$\begin{array}{c} h\left(T,P\right)=\underset{t\in T}{\max}\left\{\underset{p\in P}{\min}\left[d\left(t,p\right)\right]\right\} \end{array}$$

where *T* denotes the set of ground truth and *P* denotes the set of prediction tumor. *d*(*t*, *p*) is any metric between these points, and Euclidian distance is used in this study.

### 3.3. Segmentation Performance Analysis

**Figure 3:**
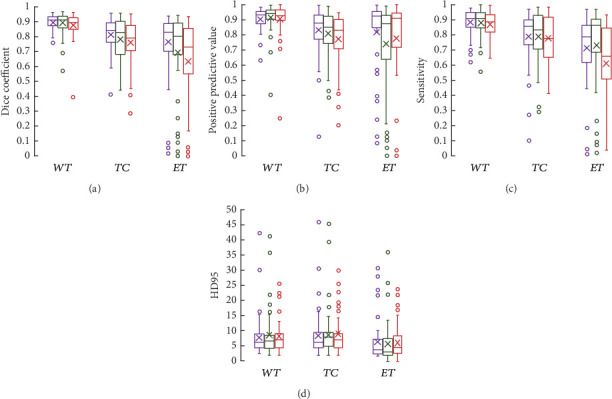
Evaluation results of experimental validation dataset for BraTS2021. Among them, (a) and (c) show the values of the Dice similarity coefficient and sensitivity; (b) and (d) show the values of the positive predictive value and Hausdorff distance (95%). Purple, green, and red represent CAGs + AdjContext + UNet (CAGs-Net), AdjContext + UNet, and UNet, respectively. WT is whole tumor, TC is tumor core, and ET is an enhancement tumor. The fork represents the mean, the horizontal line in the box represents the median, and the circle indicates outliers.

Because NCR and NET are small regions and ED and ET are highly variable regions, the segmentation of brain tumor lesions has become a challenging task. The global context feature is obtained layer by layer through AdjContext to provide the global appearance feature and semantic feature for CAGs. As shown in Figure 3, the segmentation scores of CAGs-Net (DSC, sensitivity, and 95HD) are significantly improved compared with AdjContext + UNet and UNet, and AdjContext also improves the segmentation accuracy of basic UNet. Meanwhile, the attention feature map extracted from the output layer of the CAG reveals the characteristic attention of CAGs in different modes, and CAGs strengthen the boundary of each tumor subregion by combining semantic features and appearance features of deep abstraction.

**Figure 4:**
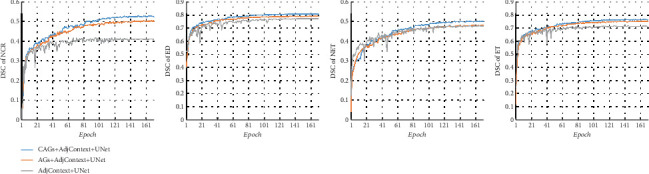
DSC of different tumor lesion areas during training, where NCR refers to the necrotic area, ED refers to the edema/inversion tissue area, NET refers to the nonenhanced tumor area, and ET refers to the enhanced tumor area. The DSC is the accuracy of the validation set in the training of three models (CAGs + AdjContext + UNet (CAGs-Net), AGs + AdjContext + UNet, and AdjContext + UNet), which can reflect the convergence state of the model in the training process.

Based on the same AdjContext + UNet network structure (see Figure 4), CAGs have a significantly better segmentation effect than AGs on each highly variable aspect of the tumor, especially for NCR and ED, while AGs have no significant improvement on NET, which also indicates that the channel-assisted regional response can improve the accuracy of tumor segmentation and the segmentation accuracy of the model. CAGs and AGs drive the convergence of the model to be relatively stable during the training process. However, AdjContext + UNet without CAGs and AGs will oscillate. It also indicates that the voxel attention response can guarantee the stability of the model convergence. Furthermore, CAGs not only make the stability of model training converge but also provide an adaptive channel classification for regional response, which alleviates the class imbalance for the segmentation of brain tumors to some extent.

Compared with traditional 3D UNet, the proposed model has a significant effect on the segmentation of glioma lesions, and it is also competitive with the other segmentation methods.  Table 1 summarizes the results of other methods of brain tumor segmentation models used for comparison. Since these models are trained on the same distribution training dataset (all on BraTS2021), the attention model can be compared with other models by comparing relevant works of literature. It is important to note that CAGs-Net does not use postprocessing (such as CRF), but it improves the segmentation results compared with the composite model–based segmentation framework, such as the DeepMedic proposed by Kamnitsas, which integrates CRF and CNN as a segmentation model. As shown in Table 1, compared with the performance of the other models, the proposed model achieved significant improvement in DSC, sensitivity, and 95HD (all in *p* < 0.05). For example, compared with Zhao et al. [12], the DSC of CAGs-Net increased by 6% for WT, 12% for TC, and 19% for ET. The 95HD of it decreased by 0.83 mm for WT, 1.75 mm for TC, and 1.78 mm for ET. Compared with AGs-Net, CAGs-Net increased by 1% for WT, 7% for TC, and 12% for ET on DSC and decreased by 0.14 mm for WT, 0.54 mm for TC, and 0.51 mm for ET on 95HD. Compared with MAG-Net, our model improved by 2% for WT, 9% for TC, and 11% for ET on DSC and decreased by 0.05 mm for WT, 0.85 mm for TC, and 0.6 mm for ET on 95HD. The CAGs can improve the segmentation accuracy of each tumor with high variability, especially in TC and ET. CAGs can improve the accuracy of tumor segmentation. The prediction of tumor segmentation illustrates that CAGs increase sensitivity and decrease edge distance by improving the ability of the dense segmentation model, which is due to the reason that the regional response classification of the channels can improve the dimension of feature fusion and improve the discrimination of features.

**Figure 5:**
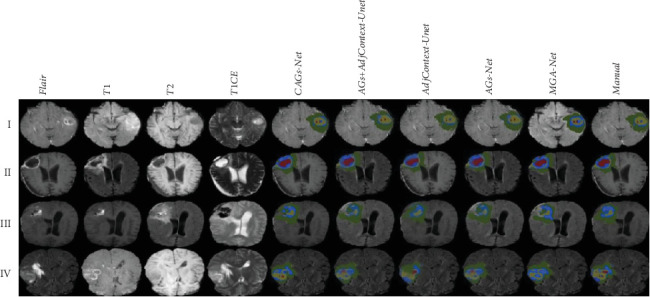
Visualization of 2D slices of 3D tumor lesion segmentation results. In the figure, Flair, T1, T1CE, and T2 show the presentation form of the tumor lesions in the image, which are combined to produce the final markers of the tumor subregion: edema (green), necrotic/cystic nuclei (yellow), nonenhancing tumor (blue), and enhancing tumor (red).

The qualitative segmentation results are shown in Figure 5. The interweaving of different lesion layers leads to the complexity of tumor segmentation. CAGs-Net, AdjContext-UNet, AGs + AdjContext-UNet, AGs-Net, and MAG-Net can accurately segment the larger tumor nuclei (see the images of Group I). However, the segmentation performance of the five models in the subregion of lesions is also different. When the four modal images provide clear subregion boundary morphology (see the images of Group II), the effect of the five segmentation models is consistent with that of manual segmentation. Under the condition of blurred boundary morphology of tumor subregions (see the images of Groups III and IV), CAGs-Net has a better segmentation effect than those of AGs + AdjContext-UNet, AGs-Net, and MAG-Net, especially for NCR and NET, although there is no obvious difference compared with AdjContext-UNet. In short, CAGs-Net can overcome the defect of the fuzzy subregion boundary compared with the other models.

To verify the effectiveness of each improved module, we further conducted ablation experiments and successively added the AdjContext module and the CAG module on the basis of UNet to compare the segmentation performance.  Table 2 presents the dice segmentation performance evaluation indicators under each configuration. It can be seen from the results that the introduction of AdjContext and the CAG module effectively improves the segmentation performance of the overall model.

## 4. Conclusion and Discussion

In this paper, we proposed a novel CAGs-Net by integrating the CAG into an adjacent-context network to tackle the persistent difficulties in segmenting small glioma subregions with indistinct margins and shape fluctuations, which uniquely combines adjacent-context modeling with CAGs to enhance feature refinement, outperforming traditional UNet-based methods. It not only can strategically combine fine-grained spatial details with abstract semantic contexts to produce distinctive hierarchical representations but also can suppress irrelevant noise from confounding regions to identify prominent image regions and magnify their effects. The experimental results on the BraTS2021 dataset demonstrated that CAGs-Net significantly improved the segmentation accuracy of tumors compared with the current segmentation methods.

Among them, how to accurately and robustly segment tumor lesions in glioma MRI images remains a great challenge, due to the blurring of the boundaries of the lesions in gliomas. When NCR and NET lesions in glioma MRI images have blurred boundaries, these features often fail to accurately delineate the boundaries of these lesions. Meanwhile, the traditional attention mechanism cannot consider the direction of feature refinement so that the small regions with high variability cannot be distinguished. A significant advantage of DNN is that it can generate multilevel features such as semantic richness and fine information. However, how to use the complementary advantages of multilevel features and adapt to learn more distinguishing features for image segmentation is still the key problem to be solved.

Therefore, we proposed an adjacent-context structure based on the residual model to form global correlation information layer by layer. Furthermore, the CAG was used to adaptively integrate appearance features and global correlation information, and this attention method refined and fused multilevel features through the dual attention of voxel space and channel, which aimed to recalibrate the characteristic response of the voxel space direction. The experimental results proved that the performance of the CAG was better than that of the attention gate and dual self-attention. Of course, whether the CAG can be used in other image segmentation is the direction of our future work.

Furthermore, we employed the strategy of combining cross-entropy loss and GDL for segmentation network training, aiming at the fuzzy boundary problem of the tumor and class imbalance for segmentation. The hybrid loss is beneficial to balance the boundary shape similarity and global shape similarity so that it can avoid the class imbalance problem of segmentation at a certain level. However, the loss function remains a great potential to avoid class imbalance in other image segmentation.

Although CAGs-Net makes full use of multilayer feature mapping differences to refine and integrate semantic information and detailed features based on CAG to improve the segmentation precisions of small lesions with large shape variability to some extent, segmentation improvements are not uniformly distributed across all tumor subregions. Therefore, how to enhance the performance consistency across different lesion types remains to be improved. Furthermore, compared with models such as 3D UNet, DeepMedic, and AGs-Net, how to further consider three-dimensional space modeling, multiscale small target detection, and improving feature selectivity on the basis of the existing models also needs further research in the future.

## Figures and Tables

**Table 1:** Performance comparison of BraTS2021 with the latest segmentation methods. The bold data indicates that our proposed method can significantly improve its conventional counterpart based on the Wilcoxon signed rank test (*p* < 0.05).

**Method**	**DSC**			**PPV**			**Sensitivity**			**95HD (mm)**		
	**WT**	**TC**	**ET**	**WT**	**TC**	**ET**	**WT**	**TC**	**ET**	**WT**	**TC**	**ET**
Kamnitsas et al. [10]	0.85	0.67	0.63	0.85	0.86	0.63	0.88	0.61	0.66	8.13	8.14	7.67
Zhao et al. [12]	0.84	0.73	0.62	0.89	0.76	0.63	0.82	0.76	0.67	8.01	8.23	7.45
Isensee et al. [13]	0.85	0.74	0.64	0.93	0.80	0.63	0.91	0.73	0.72	7.79	7.78	6.85
Pian et al. [15]	0.87	0.75	0.64	0.87	0.81	0.81	0.89	0.75	0.72	7.63	7.32	6.22
AGs-Net [17]	0.89	0.78	0.69	0.88	0.80	0.80	0.90	0.74	0.71	7.32	7.02	6.18
MGA-Net [19]	0.88	0.76	0.70	0.87	0.79	0.78	0.89	0.76	0.74	7.23	7.33	6.27
3DUNet [14]	0.86	0.73	0.63	0.82	0.81	0.64	0.89	0.75	0.73	7.82	7.99	7.11
AdjContext-UNet	0.88	0.86	**0.84**	**0.91**	0.83	0.79	**0.91**	0.76	0.71	7.17	7.28	6.45
AGs + AdjContext-UNet	0.89	0.80	0.73	0.90	0.79	0.80	0.90	0.77	0.72	7.35	6.92	6.03
CAGs-Net	**0.90**	**0.85**	0.81	0.91	**0.90**	**0.93**	0.82	**0.83**	**0.73**	**7.18**	**6.48**	**5.67**

**Table 2:** The results of ablation experiments.

	**UNet**	**UNet + AdjContext**	**UNet + CAG**	**CAGs-Net**
WT_DSC	0.75	0.78	0.75	0.76
TC_DSC	0.86	0.86	0.83	0.85
ET_DSC	0.83	0.84	0.83	0.81

## Data Availability

The data that support the findings of this study are openly available in MICCAI at http://www.braintumorsegmentation.org.
